# Clinical Outcomes and Risk Factors Associated with Spinal Kyphotic Deformity Following Osteoporotic Vertebral Fracture

**DOI:** 10.3390/jcm14082769

**Published:** 2025-04-17

**Authors:** Hayato Oishi, Keishi Maruo, Tomoyuki Kusukawa, Tetsuto Yamaura, Kazuma Nagao, Masakazu Toi, Masaru Hatano, Fumihiro Arizumi, Norichika Yoshie, Toshiya Tachibana

**Affiliations:** 1 Department of Orthopaedic Surgery, Hyogo Medical University, 1-1 Mukogawa-cho, Nishinomiya 573-1191, Hyogo, Japan; kmaruo125@gmail.com (K.M.); kkty12666@icloud.com (T.K.); tetsuto.yamaura@icloud.com (T.Y.); gaogakazu@gmail.com (K.N.); m.toi.19890908@gmail.com (M.T.); m.hatano.108@gmail.com (M.H.); f_ali_14@yahoo.co.jp (F.A.); qq.ort.yoshie@gmail.com (N.Y.); tachi@hyo-med.ac.jp (T.T.); 2 Department of Orthopaedic Surgery, Miyoshi Hospital, 24-9, Koshien-guchi-kitamachi, Nishinomiya 663-8112, Hyogo, Japan; 3 Department of Orthopaedic Surgery, Harima Hospital, 2-1-15, Harima-cho 675-0158, Hyogo, Japan; 4 Department of Orthopaedic Surgery, Gohshi Hospital, 1-8-20 Nagasu-Nishidori, Amagasaki 660-0807, Hyogo, Japan; 5 Department of Orthopaedic Surgery, Hyogo Medical University, Sasayama Medical Center, 5 Kurooka, Tanba-Sasayama 669-2321, Hyogo, Japan; 6 Department of Orthopaedic Surgery, Takarazuka City Hospital, 4-5-1, Obama 4-chome, Takarazuka 665-0827, Hyogo, Japan; 7 Department of Orthopaedic Surgery, Daiwa Central Hospital, 1-2-7 Nagahashi, Nishinari-ku, Osaka 557-0025, Osaka, Japan; 8 Department of Orthopaedic Surgery, Osaka Minato Central Hospital, 1-7-1 Isoro, Minato-ku, Osaka 552-0003, Osaka, Japan

**Keywords:** osteoporotic vertebral fractures (OVFs), global sagittal alignment (GSA), kyphotic deformity

## Abstract

**Background:** Osteoporotic vertebral fractures (OVFs) often lead to poor global sagittal alignment (GSA) and reduced quality of life (QOL). While pseudarthrosis and kyphotic deformities are well-known predictors of conservative treatment failure, the impact of vertebral collapse, paraspinal muscle degeneration, sarcopenia, and nutritional status on GSA remains unclear. This study investigated the relationship between these factors and GSA in patients with conservatively treated OVFs. **Methods:** This post hoc analysis of a multicenter prospective observational study included 70 patients (single OVF; age ≥ 60 years; 12-month follow-up). Radiographic parameters, paraspinal muscle degeneration, and nutritional status were assessed. GSA was categorized based on the sagittal vertical axis (SVA [mm]): normal (SVA ≤ 40), moderate (40 ≤ SVA ≤ 95), and severe (SVA > 95). Clinical outcomes were assessed using the Japanese Orthopaedic Association Back Pain Evaluation Questionnaire (JOABPEQ), Oswestry Disability Index (ODI), and visual analog scale (VAS). **Results:** At 12 months, 22.9% of patients had severe GSA and showed significantly lower JOABPEQ gait dysfunction scores (*p* = 0.01), higher ODI scores (*p* < 0.01), and reduced lower lumbar lordosis (*p* = 0.01). A higher prevalence of lower lumbar OVFs, including prior fractures, was observed in the severe group. No significant correlations were found between GSA and paraspinal muscle degeneration or nutritional status. **Conclusions:** OVFs in the lower lumbar spine significantly contributed to GSA deterioration. This indicates their critical role in sagittal alignment. Although paraspinal muscle degeneration and malnutrition are common in OVFs, their direct impact on GSA is limited. These findings highlight the need for targeted strategies to manage lumbar OVFs and prevent sagittal malalignment.

## 1. Introduction

Osteoporotic vertebral fractures (OVFs) typically show favorable outcomes when managed conservatively with bed rest, bracing, and anti-osteoporotic medications [1,2,3]. However, pseudarthrosis, kyphotic deformity, and vertebral collapse have been recognized as key predictors of conservative treatment failure in OVFs [4]. Pseudarthrosis is a well-documented condition, and the effectiveness of balloon has been demonstrated in patients with poor prognoses [5]. On the other hand, kyphotic deformities and vertebral collapse are expected to have lasting effects on spinal alignment; however, their clinical outcomes remain poorly understood. The etiology of kyphotic deformity after OVF is multifactorial. It includes the degree of vertebral collapse, pseudoarthrosis, and compensatory postural changes. Fusini et al. demonstrated that the morphology of vertebral fractures, particularly in the lumbar spine, significantly influences the risk of kyphosis development following conservative treatment [6]. Additionally, Prost et al. emphasized that vertebral instability, delayed union, and sagittal imbalance contribute to kyphotic progression after OVFs, and they discussed the role of both conservative and surgical strategies in managing these complications [7]. OVFs are a significant clinical concern because of their association with global sagittal malalignment and reduced quality of life (QOL) scores, accompanied by increased thoracic kyphosis (TK), pelvic tilt (PT), mismatch between pelvic incidence and lumbar lordosis (PI-LL), and sagittal vertical axis (SVA) [8]. Paraspinal muscle degeneration, characterized by a reduced cross-sectional area (CSA) and increased fatty infiltration (FI), has been implicated in the occurrence and recurrence of OVFs [9]. The FI has been identified as a predictor of vertebral collapse [10]. Despite these associations, the effects of paraspinal muscle degeneration on the global sagittal alignment (GSA) are not well understood. In addition, sarcopenia and malnutrition, frequently observed in older patients with OVFs, are closely interrelated [11]. Although malnutrition is a strong predictor of sarcopenia, their combined effect on GSA remains unclear. This study aimed to investigate the relationship between GSA and OVFs, focusing on the potential role of vertebral kyphotic deformity, progression of vertebral collapse, pseudoarthrosis, paraspinal muscle degeneration, sarcopenia, and nutritional status.

## 2. Materials and Methods

### 2.1. Study Design and Patient Selection

This was a post hoc analysis of a multicenter prospective observational study of conservatively treated OVF [12]. Eight hospitals participated in this study between May 2020 and May 2022. The inclusion criteria were as follows: (1) age ≥ 60 years; (2) OVF diagnosed within 3 weeks; (3) single OVF; (4) 12-month follow-up; and (5) whole spine X-ray assessment available at 12 months. The exclusion criteria were as follows: (1) pathological vertebral fracture; (2) spinal infection; (3) required surgery; (4) prior OVFs (>3 vertebrae); (5) subsequent OVFs during follow-up; (6) incomplete radiographic data or questionnaire; and (7) death. Prior OVFs were defined as OVFs without signal changes on the initial MRI. This study was approved by the Institutional Review Board (IRB No.3562), and informed consent was obtained from all participants.

### 2.2. Conservative Treatment

Bed rest was maintained for approximately 1–2 weeks until a customized brace was prepared. The type of brace (hard or soft) and medication for osteoporosis were determined by the attending physician depending on the patient’s age, compliance with brace wear, and ADLs prior to OVF injury. Braces were used for at least three months.

### 2.3. Patient Characteristics and Radiographic and Bone Quality Assessment

Patient-related and radiographic data were obtained from the electronic medical records. Demographic and laboratory data included patient background factors, such as age, sex, body mass index (BMI), and bone mineral density (BMD). Factors related to OVF included fracture location (T; T4-9, TL [thoracolumbar]; T10-L2, L; L3-5), number of prior OVFs, vertebral collapse (semi-quantitative [SQ] grade), wedge angle, and presence of pseudarthrosis. Radiographic parameters included TK, thoracolumbar kyphosis (TLK), LL, lower LL (LLL), PI, PT, and SVA. GSA was classified into three groups based on the Schwab classification: SVA < 40 mm (Normal; group N), 40 mm ≤ SVA ≤ 95 mm (Moderate; group M), and SVA > 95 mm (Severe; group S).

### 2.4. Paraspinal Muscle and Psoas Muscle Assessment

The lumbar indentation value (LIV) and Goutallier classification at L4–L5 were assessed using MRI to obtain quantitative and qualitative analyses of paraspinal muscle degeneration. LIV was defined as the distance between the tips of the bilateral paraspinal muscles and the tips of the spinous processes [13]. The Goutallier classification categorizes fatty degeneration into five stages: no fatty degeneration, stage 0; minimal fatty degeneration, stage 1; more muscle than fat, stage 2; equal muscle and fat composition, stage 3; and less muscle than fat, stage 4. Patients classified as having stage 3 or 4 were considered to have severe fatty degeneration. Sarcopenia was evaluated using the psoas muscle index (PMI), which was calculated as the average bilateral psoas muscle area at the L3 vertebral level divided by the square of the patient’s height, as measured by CT.

### 2.5. Patient-Reported Outcomes Measures

The Japanese Orthopaedic Association Back Pain Evaluation Questionnaire (JOABPEQ) [14], visual analog scale (VAS) score for low back pain, and Oswestry Disability Index (ODI) were used to assess clinical outcomes. The questionnaires were completed at 12 months.

### 2.6. Data Analysis

Baseline patient characteristics, muscle-related factors, OVF-related factors, radiographic parameters, and clinical outcome measures at 12 months were compared across the three groups using analysis of variance (ANOVA) [15] and the Tukey–Kramer test for continuous variables [16] and the chi-square test for categorical variables. Statistical analyses were performed using JMP version 16 (SAS Institute, Cary, NC, USA). All tests were two-tailed, and *p*-values < 0.05 were considered statistically significant.

## 3. Results

### 3.1. GSA at 12 Months After OVF

**Figure 1:**
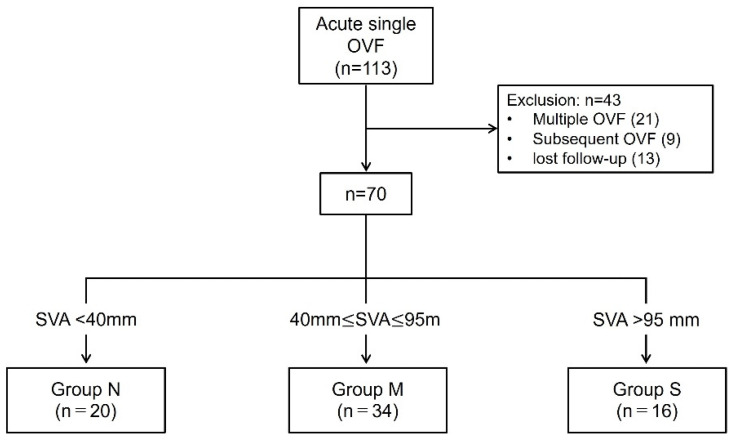
Schematic of the patient enrollment and study. Of the 113 patients, 43 were excluded. Finally, 70 patients were analyzed. They were divided into three groups based on SVA at 12-month follow-up.

A total of 113 patients with acute OVF were enrolled; 43 patients were excluded, 21 had multiple OVFs, 9 had subsequent OVF, and 13 were lost to follow-up. There were no patients who refused to participate in the final clinical assessment. Finally, 70 patients met the inclusion criteria. These patients were divided into the following three groups based on SVA at 12 months: Group N (SVA ≤ 40 mm, *n* = 20), Group M (40 mm ≤ SVA ≤ 95 mm, *n* = 34), Group S (SVA > 95 mm, *n* = 16) (Figure 1). Severe GSA was observed in 22.9% of patients 1 year (Group S) after OVF.

### 3.2. Comparison of Patient Characteristics, Muscle Assessment, and Nutrition Status Among the Three Groups at Baseline

At baseline, patients in Group N were significantly younger than those in Group M; however, no significant differences were observed in sex, BMI, or BMD (Table 1). There were no significant differences in SQ grade, prior OVF, wedge angle, or pseudarthrosis (Table 2). In terms of muscle-related factors, the Goutallier classification appeared more frequently in the S group (21% vs. 39% vs. 50%, *p* = 0.19), whereas the LIV and PMI showed no significant differences (Table 3). There were no significant differences in nutritional status among the three groups.

### 3.3. Patient-Reported Outcomes at 12 Months

Clinical outcomes were significantly lower for JOABPEQ gait dysfunction (74.9 vs. 59.7 vs. 41.2, *p* = 0.01) in Group S, and significantly higher for ODI (19 vs. 23.9 vs. 41.7, *p* < 0.01) in Group S. However, no significant differences were observed in other pain-related disabilities, lumbar spine dysfunction, psychological disability, social life disability, or VAS scores for low back pain (Table 4).

### 3.4. Radiographic Parameters at 12 Months

Radiographic parameters revealed significantly lower LLL values in Group S (33.2° vs. 26.9° vs. 23.3°, *p* = 0.01). No significant differences were identified in the TK, TLK, LL, PI, PT, or PI-LL levels (Table 5). Given that lumbar OVFs tended to be more frequent in Group S (Group N, 10%; Group M, 18%; Group S, 44%), a secondary analysis incorporating prior OVF was conducted. No significant differences were found between acute and prior OVF. However, the incidence of lumbar OVFs, including both acute and prior OVFs, was significantly higher in Group S (Table 6).

## 4. Discussion

The findings of this study revealed that 22.9% of patients with a single OVF had severe global sagittal malalignment following conservative treatment at one year. The clinical outcomes in Group S demonstrated reduced walking ability and a higher ODI. Additionally, Group S showed a significantly lower LLL and a higher incidence of lumbar OVFs, including prior OVFs, at L3–L5. No correlation was identified between GSA and the quality or quantity of the paraspinal muscles, sarcopenia, nutritional status, degree of local kyphosis, or vertebral collapse. These findings suggest that the OVFs of the lower lumbar spine are most strongly associated with the GSA.

**Figure 2:**
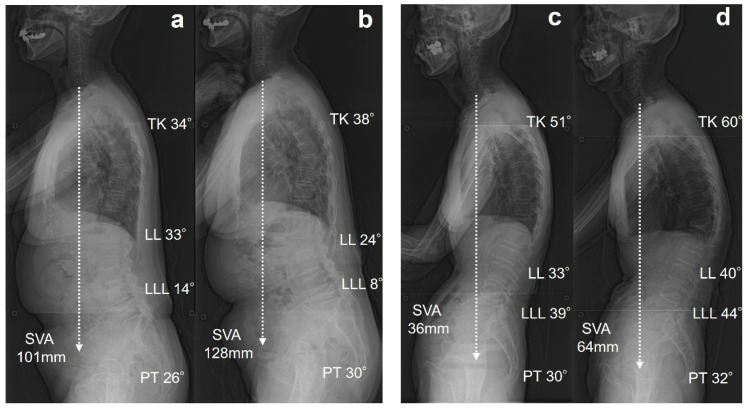
Representative two cases of single OVF at L5 and T12. (**a**) An 84-year-old woman with L5 OVF at baseline. (**b**) After one year, radiographs showed deterioration of SVA, and the patient was classified into group S. (**c**) A 72-year-old woman with T12 OVF. (**d**) After one year, radiographs showed deterioration of SVA, and the patient was classified into group M.

The presence of OVFs has been reported to correlate with poorer GSA and reduced quality of life compared with patients without OVFs [8,17,18,19,20]. A recent meta-analysis highlighted that patients with OVFs exhibit increased PT, TK, PI-LL, and SVA [20]. In the present study, although no significant differences were observed among the three groups, group S demonstrated a tendency toward greater increases in TK, PT, and PI-LL than groups N and M. Finite element model of T12 OVF have shown that as the degree of wedge deformity increases, the stress on the mid-thoracic spine and adjacent vertebrae also increases [21]. Similarly, for lower lumbar OVFs, it can be predicted that mechanical stress will be redistributed to more cranial vertebrae, potentially contributing to progressive deformity and sagittal malalignment. Pelvic retroversion and knee flexion are key adaptations to maintain horizontal gaze and postural stability in spinal deformity following OVFs [22]. These compensatory strategies are critical, particularly in elderly patients with compromised spinal support, and should be considered in the comprehensive assessment of sagittal alignment. Few studies have specifically examined the relationship between spinal alignment and OVF location. Yokoyama et al. demonstrated that new OVFs resulted in an average increase of 2.8 cm in SVA, with the lower lumbar spine being at a greater risk for sagittal alignment deterioration [23]. Plais et al. identified two key risk factors contributing to sagittal malalignment in patients with OVF: OVF localized to the lumbar region, and multiple OVFs affecting the thoracolumbar or lumbar regions [19]. Consistent with these findings, our results demonstrated a significantly reduced LLL and a higher incidence of lumbar OVF. The lower lumbar spine plays a critical role, accounting for approximately two-thirds of the LL. Therefore, lower lumbar OVFs are the most significant contributors to GSA deterioration. A typical case of Group S is shown in Figure 2a,b. An 84-year-old woman with an L5 OVF was classified as having SQ grade 2. After one year, radiographic parameters showed GSA deterioration, accompanied by the progression of vertebral collapse from grade 2 to 3. Figure 2c,d show a representative case of Group M. A 72-year-old woman had a T12 OVF with an SQ grade 1 wedge deformity at baseline. After one year, the progression of the vertebral collapse led to severe wedge deformity. While the SVA worsened, the GSA was maintained through compensatory mechanisms, including pelvic retroversion and the lower lumbar spine.

The paraspinal muscles, including multifidus and psoas major, play a significant role in the sagittal imbalance cascade [24]. Paraspinal muscle degeneration, characterized by a reduction in CSA and increased FI, is associated with the occurrence and recurrence of OVFs [9]. FI has been identified as a predictor of vertebral collapse [10]. LIV was introduced as a simple and quick measurement technique, demonstrating a strong correlation with the CSA of the paraspinal muscles [13]. Tamai et al. reported a positive correlation between LIV and LL [25]. Although we hypothesized that paraspinal muscle degeneration was associated with GSA, no significant correlation was observed. Fatty degeneration has been associated with the development of domino OVFs [12]. This study excluded cases involving multiple acute OVFs, potentially diminishing their impact on GSA. Recent studies have emphasized the significance of incorporating functional assessments, such as electromyographic analysis or MRI with muscle perfusion, to better understand the interplay between muscle quality, sarcopenia, and spinal alignment [26,27]. Due to the retrospective nature of our study and the absence of such data in our cohort, we were unable to include these evaluations. Future prospective studies incorporating functional imaging and electrophysiological measurements are warranted to provide a more comprehensive understanding of the relationship between muscle degeneration and the progression of sagittal malalignment.

Although our study did not find a statistically significant correlation between the assessed nutritional status and GSA, this may be attributed to limitations in the sensitivity of the nutritional marker used. Subclinical malnutrition may still exert a considerable impact on musculoskeletal integrity, particularly in elderly patients with osteoporosis. Future research incorporating more sensitive and comprehensive nutritional evaluations such as serum biomarkers of bone and protein turnover and whole-DEXA to assess muscle and fat mass may reveal stronger associations and provide deeper insight into the pathophysiology of sagittal malalignment. Our findings also highlight the key role of lower lumbar OVFs in the progression of sagittal imbalance. Clinically, this emphasizes the need for early recognition and intervention in patients with such fractures. Preventive and therapeutic strategies may include the use of specific spinal orthoses designed to support posture and offload the anterior spinal column, as well as rehabilitation programs focused on selective strengthening of the trunk extensor muscles. Furthermore, novel anabolic agents targeting bone remodeling may help prevent further vertebral collapse and deformity [28], potentially altering the natural history of sagittal malalignment in this patient population.

Sarcopenia is linked to increased SVA and thoracic kyphosis, especially in patients with spinopelvic mismatch [29]. This relationship indicates that sarcopenia affects the compensatory mechanisms for spinal malalignment, resulting in greater postural imbalance. Recent studies have reported that PMI correlates with the total volume of trunk muscle mass and serves as a reliable indicator of sarcopenia [30]. No significant association was identified between the PMI and GSA. Sarcopenia and malnutrition are closely interconnected conditions frequently observed in older patients with OVFs. Malnutrition is a strong predictor of sarcopenia, and studies have shown significant overlap between these conditions [11]. However, our findings revealed no significant association between nutritional status and GSA after OVF.

### Limitations

This study had several limitations. First, the present study lacks a control group and has a relatively brief follow-up duration of one year. Although a one-year follow-up is adequate for evaluating bone healing following OVF, it may be insufficient to fully elucidate the long-term progression of GSA. The baseline GSA was not assessed because most patients were unable to undergo standing radiographic evaluation at the time of injury. Consequently, it is challenging to determine whether the global sagittal malalignment observed at the one-year follow-up resulted from the OVF, or if it was due to preexisting sagittal malalignment. Second, to focus on the impact of a single OVF on GSA, this study excluded cases of acute multiple and subsequent OVFs; however, prior OVFs were included in the analysis. Excluding patients with prior OVFs and comparing them with controls would be preferable as a study design. Third, it is important to note that our method of assessing nutritional status may not have been sufficiently sensitive to detect subtle but clinically relevant variations. This limitation may have contributed to the observed lack of correlation between nutritional status and GSA. More refined tools, including biochemical markers and comprehensive body composition analysis, should be considered in future studies to better capture the influence of nutritional factors on spinal alignment. Nonetheless, the strength of this study is its comprehensive assessment, which included not only the degree of vertebral collapse and kyphotic deformity associated with OVFs, but also assessments of paraspinal muscles, sarcopenia, and nutritional status.

## 5. Conclusions

OVFs occurring in the lower lumbar spine significantly contribute to the deterioration of GSA, highlighting their role in the progression of sagittal malalignment. These findings underscore the importance of early detection and targeted interventions for lumbar OVFs to prevent long-term postural decompensation. Clinically, this highlights the need for a multidisciplinary management approach, including rigid bracing, anti-osteoporotic medication, and rehabilitation programs specifically designed to enhance paraspinal muscular support. From a research perspective, future studies should focus on longer-term follow-up, the integration of functional and nutritional assessments, and the development of individualized strategies to preserve spinal alignment and mobility in the aging population.

## Figures and Tables

**Table 1:** Baseline patient characteristics.

Variable	Group N	Group M	Group S	*p*-Value
Age (years)	76.2 ± 7.0	81.1 ± 7.7	81.8 ± 6.0	0.03 *
Sex, female (*n*, %)	15 (75)	28 (82)	14 (88)	0.6
BMI (kg/m^2^)	22.7 ± 3.2	22.6 ± 3.0	22.9 ± 3.2	0.9
Lumbar YAM (%)	77.1 ± 15.2	81.5 ± 18.2	77.4 ± 14.8	0.7
Total hip YAM (%)	71.4 ± 9.9	74.3 ± 16.2	68.8 ± 16.9	0.5

Values are presented as mean ± standard error, Group N; SVA < 40 mm, Group M; 40 ≤ SVA ≤ 95 mm, Group S; SVA > 95 mm, BMI; body mass index, YAM; young adult mean, *, *p* < 0.05.

**Table 2:** Fracture location and the severity of fracture in the three groups.

Variable	Group N	Group M	Group S	*p*-Value
Fracture location, *n* (%)				0.1
Thoracic (T4-9)	1 (5)	2 (6)	1 (6)	
TL junction (T10-L2)	17 (85)	26 (76)	8 (50)	
Lumbar (L3-5)	2 (10)	6 (18)	7 (44)	
SQ grade, *n* (%)				0.3
0	0	3 (9)	2 (13)	
1	11 (55)	12 (35)	10 (63)	
2	8 (40)	15 (44)	4 (25)	
3	1 (5)	4(12)	0	
Prior OVF, *n* (%)	5 (25)	11 (32)	9 (56)	0.1
Wedge angle (°)	15.1 ± 8.3	16.8 ± 9.2	16.7 ± 9.1	0.8
Pseudoarthrosis	5 (25)	6 (18)	6 (37.5)	0.3

Values are presented as mean ± standard error; TL, thoracolumbar; SQ, semi-quantitative.

**Table 3:** Muscle-related factors and nutritional status in the three groups.

Variable	Group N	Group M	Group S	*p*-Value
Goutallier 3 or 4, *n* (%)	4 (20)	13 (38)	8 (50)	0.2
LIV (mm)	10.9 ± 4.9	10.8 ± 5.3	8.3 ± 6.3	0.3
PMI (cm^2^)/m^2^	2.1 ± 0.6	2.0 ± 0.5	2.0 ± 0.7	0.7
CONUT score	1.7 ± 2.5	1.7 ± 1.4	2.1 ± 1.8	0.8

Values are presented as mean ± standard error; LIV, lumbar indentation value; PMI, psoas muscle index; CONUT, controlling nutritional status.

**Table 4:** Results of JOABPEQ, VAS score, and ODI at 12 months.

Variable	Group N	Group M	Group S	*p*-Value
JOABPEQ				
Pain-related disorder	68.5 ± 36.1	70.5 ± 30.1	64.2 ± 39.5	0.96
Lumbar function	71.6 ± 28.8	68.9 ± 29.5	61.8 ± 33.0	0.66
Walling ability	74.9 ± 23.9	59.7 ± 33.7	41.2 ± 33.1	0.01 *
Social life function	67.1 ± 25.5	58.4 ±23.0	49.2 ± 25.0	0.05
Mental health	61.5 ± 15.5	57.3 ± 17.7	48.0 ± 19.0	0.12
VAS for LBP	23.7 ± 4.6	30.8 ± 26.2	24.7 ± 24.5	0.34
ODI	19.0 ± 1943	23.9 ± 18.9	41.7 ± 25.1	<0.01 *

Values are presented as mean ± standard error. JOABPEQ, Japanese Orthopaedic Association Back Pain Evaluation Questionnaire; VAS, visual analog scale; LBP, low back pain; ODI, Oswestry disability index; *, *p* < 0.05.

**Table 5:** Sagittal spinal alignment at 12 months.

Variable	Group N	Group M	Group S	*p*-Value
TK (°)	31.6 ± 14.9	34.7 ± 16.0	40.9 ± 19.8	0.25
TLK (°)	20.5 ± 9.1	22.6 ± 14.4	22.5 ± 13.5	0.83
LL (°)	39.7 ± 12.4	34.4 ± 11.5	26.9 ± 23.3	0.05
LLL (°)	33.2 ± 7.0	26.9 ± 10.2	23.3 ± 12.9	0.01 *
PI (°)	54.2 ± 7.9	49.2 ± 10.2	49.7 ± 7.2	0.14
PT (°)	24.3 ± 7.2	24.6 ± 7.6	29.1 ± 17.9	0.33
PI-LL (°)	14.4 ± 12.0	14.8 ± 13.2	22.7 ± 25.1	0.24

Values are presented as mean ± standard error. Group N, SVA < 40 mm; Group M, 40 ≤ SVA ≤ 95 mm; Group S, SVA > 95 mm; TK, thoracic kyphosis (T5-12); TLK, thoracolumbar kyphosis (T10-L2); LL, Lumbar lordosis; LLL, lower lumbar lordosis (L4-S1); PI, pelvic incidence; PT, pelvic tilt; *, *p* < 0.05.

**Table 6:** The impact of lumbar OVF on global sagittal alignment.

Variable	Group N	Group M	Group S	*p*-Value
L3-5 acute OVF, *n* (%)	2 (10)	6 (18)	7 (44)	0.1
L3-5 prior OVF, *n* (%)	4 (20)	2 (6)	4 (25)	0.1
L3-5 OVF, *n* (%) (acute + prior OVF)	5 (25)	8 (24)	11 (69)	<0.01 *

Group N, SVA < 40 mm; Group M, 40 ≤ SVA ≤ 95 mm; Group S, SVA > 95 mm; *, *p* < 0.05.

## Data Availability

The datasets used and/or analyzed in the current study are available from the corresponding author upon reasonable request.

## Abbreviations

The following abbreviations are used in this manuscript:

BMD	bone mineral density
BMI	body mass index
CSA	cross-sectional area
CT	computed tomography
FI	fatty infiltration
GSA	global sagittal alignment
JOABPEQ	Japanese Orthopaedic Association Back Pain Evaluation Questionnaire
LIV	lumbar indentation value
LL	lumbar lordosis
LLL	lower lumbar lordosis
MRI	magnetic resonance imaging
ODI	Oswestry Disability Index
OVF	osteoporotic vertebra fracture
PI	pelvic incidence
PMI	psoas muscle index
PT	pelvic tilt
QOL	quality of life
SQ	semi-quantitative
SVA	sagittal vertical axis
TK	thoracic kyphosis
TL	Thoracolumbar
TLK	thoracolumbar kyphosis
VAS	visual analog scale
